# Stay-at-home orders associate with subsequent decreases in COVID-19 cases and fatalities in the United States

**DOI:** 10.1371/journal.pone.0248849

**Published:** 2021-06-10

**Authors:** James H. Fowler, Seth J. Hill, Remy Levin, Nick Obradovich

**Affiliations:** 1 Infectious Diseases and Global Public Health Division, University of California, San Diego, San Diego, CA, United States of America; 2 Political Science Department, University of California, San Diego, San Diego, CA, United States of America; 3 Economics Department, University of Connecticut, Storrs, CT, United States of America; 4 Center for Humans and Machines, Max Planck Institute for Human Development, Berlin, Germany; Frankfurt Institute for Advanced Studies, GERMANY

## Abstract

Governments issue “stay-at-home” orders to reduce the spread of contagious diseases, but the magnitude of such orders’ effectiveness remains uncertain. In the United States these orders were not coordinated at the national level during the coronavirus disease 2019 (COVID-19) pandemic, which creates an opportunity to use spatial and temporal variation to measure the policies’ effect. Here, we combine data on the timing of stay-at-home orders with daily confirmed COVID-19 cases and fatalities at the county level during the first seven weeks of the outbreak in the United States. We estimate the association between stay-at-home orders and alterations in COVID-19 cases and fatalities using a difference-in-differences design that accounts for unmeasured local variation in factors like health systems and demographics and for unmeasured temporal variation in factors like national mitigation actions and access to tests. Compared to counties that did not implement stay-at-home orders, the results show that the orders are associated with a 30.2 percent (11.0 to 45.2) average reduction in weekly incident cases after one week, a 40.0 percent (23.4 to 53.0) reduction after two weeks, and a 48.6 percent (31.1 to 61.7) reduction after three weeks. Stay-at-home orders are also associated with a 59.8 percent (18.3 to 80.2) average reduction in weekly fatalities after three weeks. These results suggest that stay-at-home orders might have reduced confirmed cases by 390,000 (170,000 to 680,000) and fatalities by 41,000 (27,000 to 59,000) within the first three weeks in localities that implemented stay-at-home orders.

## Introduction

Coronavirus disease 2019 (COVID-19) first appeared as a cluster of pneumonia cases in Wuhan, China on December 31, 2019 [1] and was declared a global pandemic by the World Health Organization (WHO) on March 11, 2020 [2]. As of May 27, 2020, the European Centers for Disease Control reports that worldwide there have been 5,555,737 confirmed cases of COVID-19, resulting in 350,212 deaths [3].

The United States has both the highest number of cases (1,681,212) and deaths (98,916) due to the disease [3]. As a result, the U.S. government has been widely criticized for inaction in the early stages of the pandemic [2]. Although the first confirmed case of COVID-19 was reported to the Centers for Disease Control on January 21, 2020 and documented transmission commenced immediately [4], a national state of emergency was not declared until nearly two months later on March 13. At that time, the only mandatory response at the national level was international travel restrictions [5].

While the national government has the authority to act, the United States is a federal political system where public health is normally the purview of the fifty states. Furthermore, each state often delegates health authority to cities and/or counties, geographic political units nested within states. As a result, responses to COVID-19 varied across states and counties and led to spatial and temporal variation in implementation of mitigation procedures. This variation in policy responses has likely contributed to significant variation in the incidence of cases and fatalities—as well as the downstream social and economic effects of the disease—across jurisdictions in the United States [6–11].

Numerous government policies have been proposed and used to mitigate the spread and consequence of pandemic diseases like COVID-19, ranging from investments in medical testing, contact tracing, and clinical management, to school closures, banning of mass gatherings, quarantines, and population stay-at-home orders [12]. China’s extensive interventions appear to have been successful at limiting the outbreak [13, 14]. These include quarantines both for those diagnosed and those undiagnosed but who had been in Hubei province during the outbreak [15], and restrictions on travel to and from affected areas [16]. In contrast, school closures across East Asia were estimated to be much less effective [17].

With estimates that a large portion of transmissions occur from pre-symptomatic and asymptomatic individuals [18], epidemiological simulations suggest that quarantines of symptomatic individuals alone will be insufficient to halt the pandemic [19]. This has led to widespread adoption of population-wide policies to dramatically reduce social contact. A crucial question of interest to public policymaking is the effectiveness of these different mitigation policies in slowing transmission rates of the virus [20, 21].

Here, we study the role of stay-at-home orders, perhaps the most common policy intervention in the United States aimed at reducing the transmission of SARS-COV-2. Stay-at-home orders require citizens to shelter in their residence with very few exceptions and have typically been implemented along with school closures, bans on mass gatherings, and closure of non-essential businesses. These policies are associated with a significant reduction in observed mobility [22], and early evidence from New York City and California suggests that they can be effective in reducing case growth in the United States [8, 23].

A variety of statistical challenges confront estimation of the effectiveness of non-pharmaceutical policy interventions on SARS-COV-2 transmission. The most significant of these is the problem of treatment endogeneity. Because policymakers implement interventions by choice, implementation might be correlated with disease progression. Population-wide interventions might be more likely to occur the more dramatic the pandemic. This “selection into treatment” problem may confound the influence of the policy intervention on disease progression with the natural course of the pandemic.

Existing work aimed at estimating the causal effects of stay-at-home orders attempts to overcome this challenge by employing an event-study design, comparing the progression of the disease over time in counties that implemented stay-at-home orders before and after the order was implemented [24]. Because such designs estimate the effect of the intervention based on within-unit changes in the outcome, they do not account for the possibility that counties that implemented stay-at-home orders may differ systematically from counties that did not. Consequently, it is possible that the effects of the policy estimated in previous work may be due to unobserved characteristics of implementing counties that correlate with disease dynamics, rather than the causal effects of the stay-at-home orders themselves.

To illustrate this issue, consider the following hypothetical scenario. Imagine that stay-at-home orders have no effect on disease progression, but that individual behavioral changes—unrelated to formal policy restrictions—do limit transmission. Suppose the March 13 declaration of a national emergency due to COVID-19 induced some counties to issue stay-at-home orders and also induced millions of Americans nationwide to—independent of orders—change their behavior from March 13 forward. Suppose we collected data on rates of transmission before and after stay-at-home orders in issuing counties, only, to conduct an event study design to estimate the effect of such orders pre-order versus post-order. We would see that SARS-COV-2 transmission rates fell following implementation of stay-at-home orders. And—absent accounting for reduced transmission due to individual behavioral changes nationwide—we would erroneously conclude that stay-at-home orders effectively and substantively changed transmission dynamics.

Identifying the effect of orders using over-time changes in counties that issued stay-at-home orders could induce the kind of bias we described in the previous paragraph. Even if these designs include never-order units in their sample, the inclusion of unit fixed effects means that units that never change policy do not contribute to the estimated effects of that policy. To overcome this identification challenge, we implement a difference-in-differences approach that uses the hundreds of counties that do not issue a stay-at-home order during our time period of study as explicit controls. We group counties by the calendar date of their issuance of stay-at-home orders. For each calendar date-of-order group, we compare case progression following the stay-at-home order to case progression in the no-stay-at-home order control counties for each calendar date subsequent to that group’s order date. Our method identifies the causal effects of stay-at-home orders only under the assumption that the disease would have progressed in implementing counties in the same way as it did in fact progress in non-implementing counties, had counties with stay-at-home orders never issued the orders.

This approach complements the event-study approach. The event-study approach is a comparison of early and late adopters. The estimand of the comparison of early and late adopters is the impact on cases and fatalities in (eventually) adopting counties if orders had been adopted earlier. Our approach, in contrast, compares adopters and non-adopters by calendar date to hold constant the national context. The estimand of this comparison is the impact on cases and fatalities in non-adopting counties if orders had been adopted there on the calendar date of comparison.

The relevant estimand depends upon the policy question of interest. However, if both designs return similar empirical results, our confidence in the relationship between stay-at-home orders and reductions in COVID-19 cases and fatalities should correspondingly increase. Our hope is that our empirical approach can serve as a complement to event-study designs such Courtemanche et al. (2020) [24], demonstrating the robustness of empirical results across varying identifying assumptions. Our figures below further highlight the importance of appropriately controlling for nationwide context.

One option for analysis is to estimate the effects of policy interventions relative to the local date of the first case. However, time since local onset is confounded with the national informational environment and corresponding individual behavioral changes. Our methodological approach is to use calendar date to control for the national stimulus on individual behavioral change.

Two other challenges to estimation should be acknowledged. First, stay-at-home orders are sometimes issued concurrently to other policy interventions such as school closures, restaurant and bar closures, and masking requirements. The estimates we present below are best interpreted as a compound treatment of stay-at-home orders plus the weighted average of the correlated interventions in our sample. Second, county-specific policies might spill over to counties geographically adjacent to treated counties, e.g. other counties in the same commuting zone. We note that positive spillovers to adjacent counties who do not issue orders would attenuate estimated effects if the adjacent counties are in the never-order control group, meaning our estimates here would be downward biased. Given our grouping strategy, such spillovers would not influence our estimated effects if adjacent counties issued orders on different calendar dates, which is a benefit of our design relative to a standard difference-in-differences approach.

## Results

**Fig 1:**
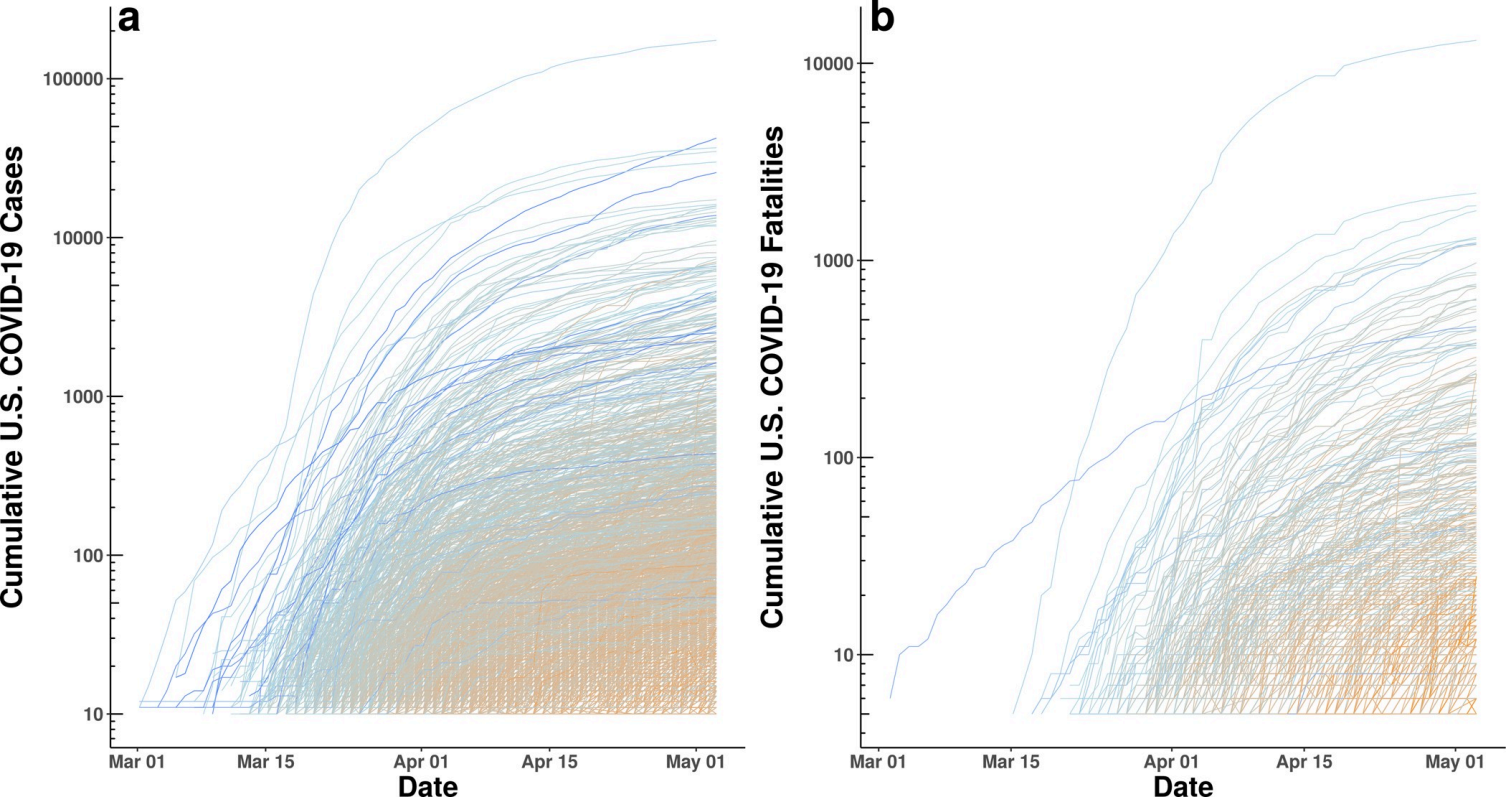
**(a)** Log of cumulative confirmed COVID-19 cases by county and date. **(b)** Log of cumulative confirmed COVID-19 fatalities by county and date. Each panel plots the time series, at the county-level, of cumulative cases and cumulative deaths. As can be seen, some of the counties with earliest detection of cases observed the highest total number of cases as well as some of the steepest growth in cases. Lines are gradient-colored by date of first case and date of first fatality, respectively (dark blue = early to orange = late).

We collected data on stay-at-home orders, COVID-19 confirmed cases, rates of testing, and fatalities by day and county throughout the United States (see Methods) from March 24th through May 7th, 2020. Fig 1 shows that the number of cases and fatalities grew exponentially from March 1 to May 3, 2020. It also suggests that efforts to “flatten the curve” initiated in mid-to-late March helped to reduce the rate of exponential growth.

**Fig 2:**
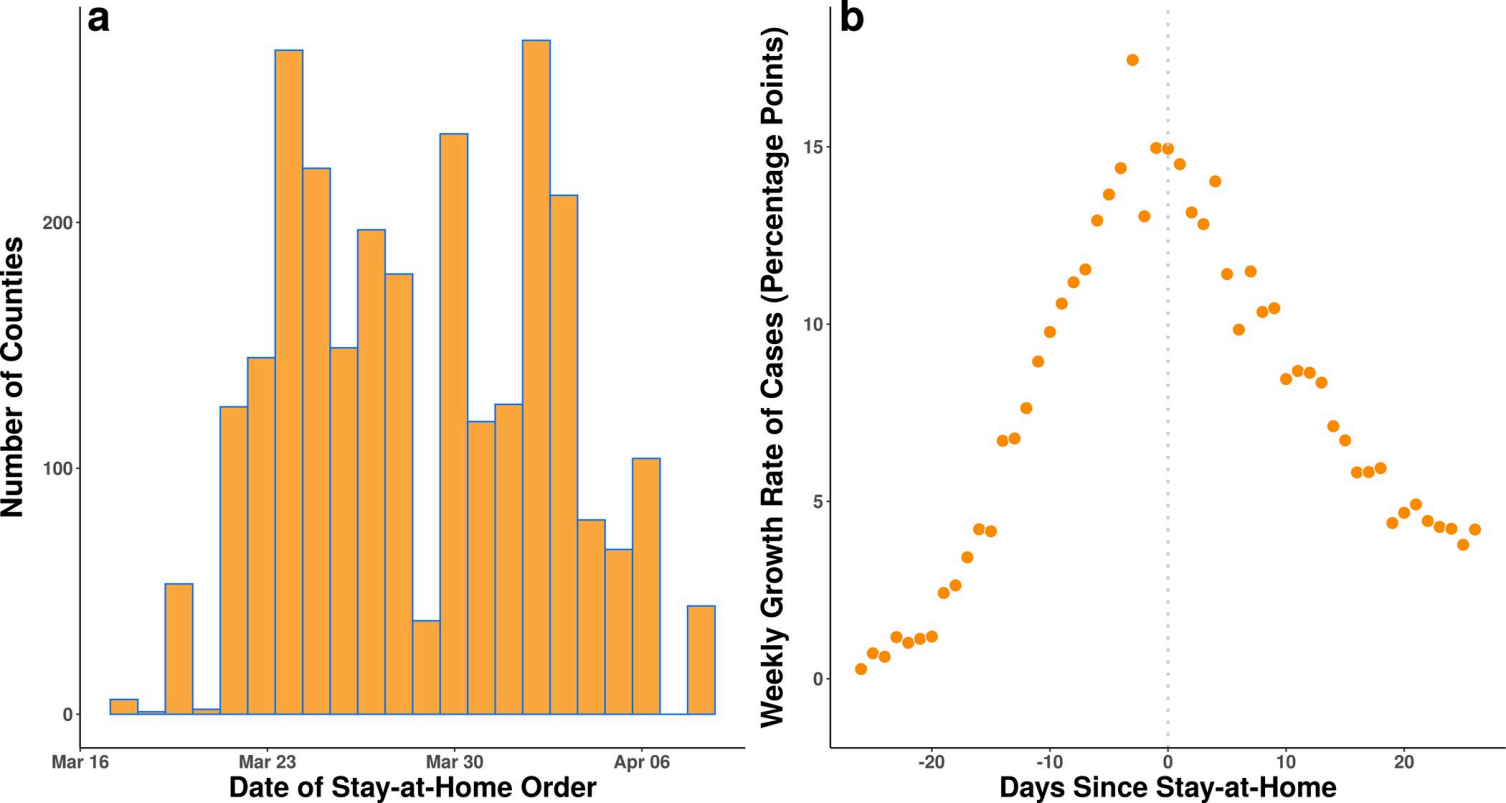
**(a)** Distribution of stay-at-home orders at the county level by date in the United States. There is substantial calendar date variation in the timing of these orders over an approximately three-week period. **(b)** Mean county-level weekly growth in total confirmed COVID-19 cases in the United States by the number of days before or after a stay-at-home order. Each point represents the weekly mean growth rate of cases for the counties with stay-at-home orders on a specific day relative to the date of that county’s stay-at-home order issuance. Counties that never had a stay-at-home order are not included in this panel. As can be seen, after stay-at-home orders, case growth declines substantially. However, without comparing to counties that never issued stay-at-home orders, this apparent decline due to stay-at-home orders may be confounded by contemporaneous changes in individual behaviors, nationally.

Fig 2A shows the distribution of stay-at-home order dates. By April 7, 2020, 18 states (1,451 counties) had different counties with different order dates, 27 states and the District of Columbia had statewide orders with no local variation (1,307 counties) and 5 states (386 counties) had no order in place. Fig 2B shows how the mean county-level weekly growth rate in COVID-19 cases changed relative to the date the stay-at-home order went into effect in counties that implemented a stay-at-home order (this depicts results similar to what would be obtained by an event study approach). If one evaluated only the information presented in Fig 2B, one might conclude that stay-at-home orders produced substantial decline in case growth. But without controlling for changes in case growth in counties with no stay-at-home orders, this conclusion might be biased either upwards or downwards in magnitude by behavioral changes correlated with orders.

**Fig 3:**
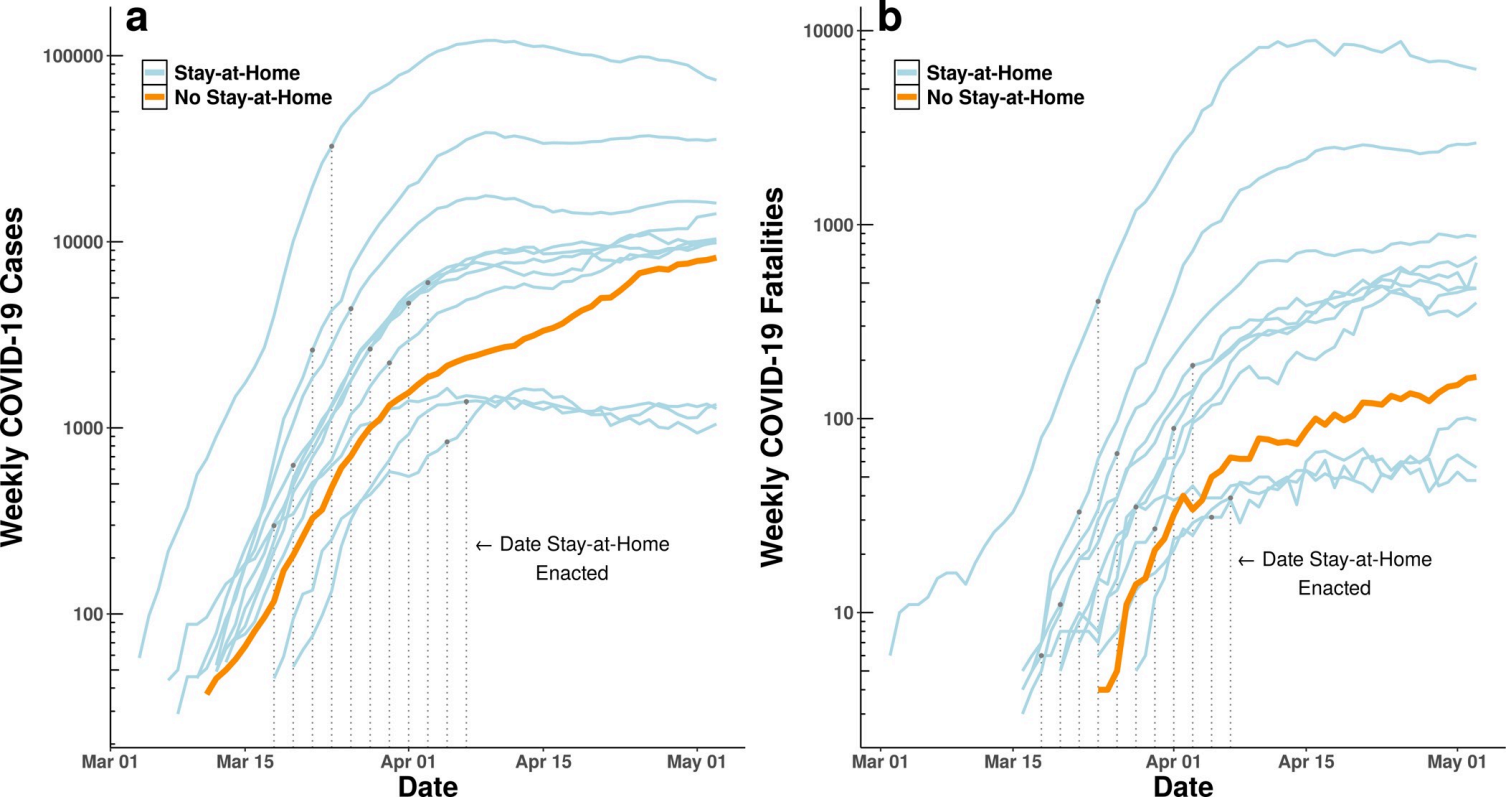
Weekly **(a)** newly-confirmed COVID-19 cases in the United States and **(b)** confirmed fatalities. Each line represents an aggregation of counties that issued stay-at-home orders during the same two-day period (blue) or did not issue an order (orange). Raw observations are grouped to two-day pairs and plotted along calendar dates to facilitate visualization of the potential for contemporaneous national trends between stay-at-home counties and counties with no stay-at-home orders. Dotted vertical lines indicate the calendar date in which each county group implemented their stay-at-home policies.

The growth rate of cases began to decline following stay-at-home orders. In Fig 3, we group counties by the date a stay-at-home order was implemented and show how confirmed cases and fatalities observationally change with respect to the order. For example, the top blue curve in both panels refers to all counties with stay-at-home orders enacted on March 23 or 24, notably including the epicenter of the pandemic in New York. We then plot the set of counties that did not issue any stay-at-home order during this time period with a thicker orange line to highlight how this group of counties differs from those with an order across the same calendar dates. Although the curves have been “flattened” in all counties, the flattening in counties without an order is notably slower, particularly with respect to cases.

Table 1 shows growth in log weekly cases both on the date an order goes into effect and 21 days after the order. Each row presents corresponding weekly growth on the same dates for the set of all counties where no such order ever went into effect. For example, the first row shows that weekly cases for the six counties who issued an order on March 17 grew from March 10 to March 17 by 1.02 units on a log scale. For the 386 counties that would not at any point in our time period issue such an order, weekly case growth from March 10 to March 17 averaged 1.39 units on a log scale. Twenty-one days after the order, case growth at stay-at-home counties had declined to 0.71 and in control counties to 1.10.

**Table 1:** Comparison of weekly changes in log daily confirmed cases of COVID-19 between 2,647 counties with stay-at-home orders and 386 counties without orders relative to the same date for each unique stay-at-home date.

		One Week Change in Log Weekly Cases						
		Counties With Stay-at-Home Order		Counties Without Stay-at-Home Order				
Date Stay-at-Home Order Went Into Effect	Number of Counties	On Day of Order	21 Days After Order	On Day of Order	21 Days After Order	21-Day Difference in Change, With Order	21-Day Difference in Change, Without Order	Difference in Difference, Counties With Orders and Without Orders
**3/17/20**	6	1.02	0.71	1.39	1.10	-0.31	-0.29	-0.02
**3/19/20**	52	2.00	1.00	1.34	0.89	-1.00	-0.45	-0.55
**3/21/20**	104	2.52	0.88	1.52	0.66	-1.64	-0.85	-0.79
**3/22/20**	23	2.87	0.99	1.59	0.66	-1.88	-0.92	-0.96
**3/23/20**	209	2.63	0.41	1.50	0.54	-2.22	-0.96	-1.26
**3/24/20**	258	2.20	0.57	1.61	0.50	-1.63	-1.10	-0.53
**3/25/20**	308	1.93	0.47	1.65	0.46	-1.46	-1.19	-0.27
**3/26/20**	77	1.67	0.22	1.42	0.39	-1.45	-1.02	-0.43
**3/27/20**	136	1.52	0.16	1.42	0.34	-1.36	-1.08	-0.28
**3/28/20**	174	1.40	0.33	1.35	0.33	-1.07	-1.02	-0.04
**3/29/20**	38	1.32	-0.10	1.23	0.25	-1.41	-0.98	-0.44
**3/30/20**	324	1.35	0.32	1.29	0.28	-1.03	-1.01	-0.02
**3/31/20**	31	1.54	0.58	1.10	0.28	-0.96	-0.82	-0.14
**4/1/20**	126	1.10	-0.13	0.93	0.31	-1.24	-0.62	-0.61
**4/2/20**	274	1.13	0.17	0.89	0.30	-0.97	-0.58	-0.38
**4/3/20**	290	0.83	0.08	0.78	0.32	-0.75	-0.46	-0.29
**4/4/20**	66	0.54	-0.04	0.66	0.38	-0.59	-0.29	-0.30
**4/6/20**	104	0.58	-0.17	0.54	0.40	-0.76	-0.14	-0.61
**4/7/20**	44	0.61	-0.05	0.50	0.46	-0.66	-0.04	-0.62

Although mean rates of change decline for all counties, the rightmost column shows that all of the 2,647 counties with orders are in groups that decline faster than counties without orders.

We group counties by the day their order went into effect to control for the national information environment. We assume individuals in all geographies respond to information about the pandemic whether or not their county issues an order. What this grouping asks is if—conditional on the national news environment on a specific calendar day—case and fatality growth patterns differ by whether the county issued a local stay-at-home order.

Although Table 1 shows that growth slowed for all counties over each 21 day period, with or without an order, the last column shows that it slowed faster for every single county group with an order than for the counties that did not issue an order. This comparison shows that while populations in all counties experienced a reduction in pandemic growth, counties that issued a stay-at-home order had growth slow more quickly than counties that did not. This explicit comparison between ‘treated’ and ‘control’ counties allows for a more explicit evaluation of the effects of the orders and provides further—and differentiated—evidence that the orders themselves correspond to changing infection patterns [24].

Fatalities follow the same pattern as cases (Table 2). Once again, growth slows for all counties, but the rightmost column shows that 1,931 of the 2,647 counties with orders are in groups that decline faster than counties without orders.

**Table 2:** Comparison of weekly changes in log daily fatalities due to COVID-19 between 2,647 counties with stay-at-home orders and 386 counties without orders relative to the same date for each unique stay-at-home date.

		One Week Change in Log Weekly Fatalities						
		Counties With Stay-at-Home Order		Counties Without Stay-at-Home Order				
Date Stay-at-Home Order Went Into Effect	Number of Counties	On Day of Order	21 Days After Order	On Day of Order	21 Days After Order	21-Day Difference in Change, With Order	21-Day Difference in Change, Without Order	Difference in Difference, Counties With Orders and Without Orders
**3/17/20**	6	1.10	0.12	-0.69	0.94	-0.98	1.63	-2.62
**3/19/20**	52	1.20	0.76	-0.69	0.43	-0.45	1.12	-1.57
**3/21/20**	104	2.56	0.82	-0.69	0.71	-1.74	1.40	-3.14
**3/22/20**	23	1.85	0.63	0.00	0.40	-1.21	0.40	-1.61
**3/23/20**	209	1.81	0.33	0.00	0.34	-1.48	0.34	-1.82
**3/24/20**	258	3.62	0.61	1.61	0.16	-3.01	-1.45	-1.56
**3/25/20**	308	2.01	0.43	1.61	0.33	-1.59	-1.28	-0.31
**3/26/20**	77	1.61	0.10	1.79	0.47	-1.51	-1.32	-0.19
**3/27/20**	136	1.10	-0.14	2.48	0.16	-1.24	-2.32	1.08
**3/28/20**	174	2.25	0.28	2.71	0.29	-1.98	-2.41	0.44
**3/29/20**	38	1.50	0.12	2.08	0.26	-1.39	-1.82	0.43
**3/30/20**	324	0.80	0.34	2.40	0.31	-0.46	-2.09	1.63
**3/31/20**	31	1.28	0.01	1.61	0.49	-1.27	-1.12	-0.14
**4/1/20**	126	1.72	0.05	1.89	0.32	-1.67	-1.57	-0.10
**4/2/20**	274	1.66	0.44	1.92	0.16	-1.22	-1.76	0.54
**4/3/20**	290	1.12	0.06	1.07	0.34	-1.06	-0.73	-0.33
**4/4/20**	66	1.86	0.27	0.96	0.18	-1.59	-0.77	-0.81
**4/6/20**	104	0.77	-0.13	0.92	0.23	-0.90	-0.69	-0.21
**4/7/20**	44	0.65	0.40	0.94	0.02	-0.26	-0.92	0.67

Although mean rates of change decline for all counties, the rightmost column shows that 1,931 of the 2,647 counties with orders are in groups that decline faster than counties without orders.

Although these results suggest that stay-at-home orders worked, one must be cautious in drawing causal inference from this approach. A number of factors might confound this association in the raw data. For example, stay-at-home orders might closely follow earlier targeted mitigation measures at the national level (such as travel restrictions issued by the State Department or recommendations by the CDC on mass gatherings). There might also exist spurious correlation between local factors (such as the date of onset of the disease or the capacity of the health system) and the timing of stay-at-home orders. To control for these factors, we apply a two-way fixed-effects difference-in-differences model to the data (see Methods).

Table 3 shows results from three models for growth in weekly confirmed cases that estimate the effect of stay-at-home orders 7 days, 14 days, and 21 days after the orders go into effect. A fourth model shows an estimate of the association between orders and growth in weekly fatalities after 21 days. All models also control for changes in the availability of testing (see Methods). The key estimates are in the top row of Table 3, and exponentiating these coefficients (*exp*(*β*) − 1) allows us to interpret them as percentage changes in weekly cases. They suggest that stay-at-home orders are associated with a 30.2 percent (10.5 to 45.6) reduction in weekly incident cases after one week, a 40.0 percent (22.9 to 53.2) reduction after two weeks, and a 48.8 percent (35.8 to 62.5) reduction after three weeks. Stay-at-home orders are also associated with a 59.8 percent (32.3 to 76.1) reduction in weekly fatalities after three weeks.

**Table 3:** Difference-in-difference weighted least squares regression results.

	*Dependent Variable: One Week Change in Log Weekly Confirmed Cases*						*Dependent Variable: One Week Change in Log Weekly Fatalities*	
	*Model 1*		*Model 2*		*Model 3*		*Model 4*	
	*7 Days After*		*14 Days After*		*21 Days After*		*21 Days After*	
	*Estimate*	*CI*	*Estimate*	*CI*	*Estimate*	*CI*	*Estimate*	*CI*
***Difference Between Counties With and Without and Order*, *After the Order***	-0.302	[-.105, -.456]	-0.400	[-.229, -.532]	-0.488	[-.358, -.625]	-0.598	[-.323, -.761]
***Mean Difference Between Counties With and Without an Order***	0.462	[.213, .763]	0.462	[.139, .877]	0.405	[.050, .880]	0.896	[.104, 2.256]
***Mean Difference Before and After the Order***	-0.213	[-.320, -.090]	-0.237	[-.441, .043]	0.209	[-.077, .585]	-0.874	[-.874, -.874]
***One Week Change in Log Weekly Tests Performed***	0.234	[-.097, .685]	0.568	[.101, 1.233]	1.340	[.823, 2.003]	-0.719	[-.911, -.117]
***Adjusted R***^***2***^	0.82		0.81		0.85		0.35	

Estimates are reported in exponentiated form (*exp*(*β*) - 1), which means that they can be interpreted as percentage changes in weekly cases. For example, a coefficient of -0.302 represents a 30.2% reduction. CI values represent 95% confidence intervals around the estimates. The first row shows the estimated difference-in-differences treatment effect of stay-at-home orders on log weekly confirmed COVID-19 cases after 7 days (Model 1), 14 days (Model 2), and 21 days, and effect on log weekly COVID-19 fatalities after 21 days (Model 4). The results in row one are the composition of two differences, across the treatment and control, and before and after the treatment was deployed. The subsequent two rows of the table provide estimates for each of the two dimensions of differencing separately. The second row depicts the mean difference between counties with and without an order (treatment vs. control). The third row depicts the mean difference before and after the order for both treatment and control counties (pre vs. post). The fourth row depicts the coefficients associated with our control variable of change in the log of weekly tests performed. Results for all models are for two observations each (one on the date of the order and one after the order) for 2,647 counties grouped by date of order (*N* = 22), and paired with two observations each for 386 counties on the same dates where no order was issued. All 88 county-group observations are weighted by the number of counties per group, fixed effects for county group and date are included in each model, and standard errors are clustered by group. These models control for all fixed factors that vary between counties and factors that vary over time at the national level.

**Fig 4:**
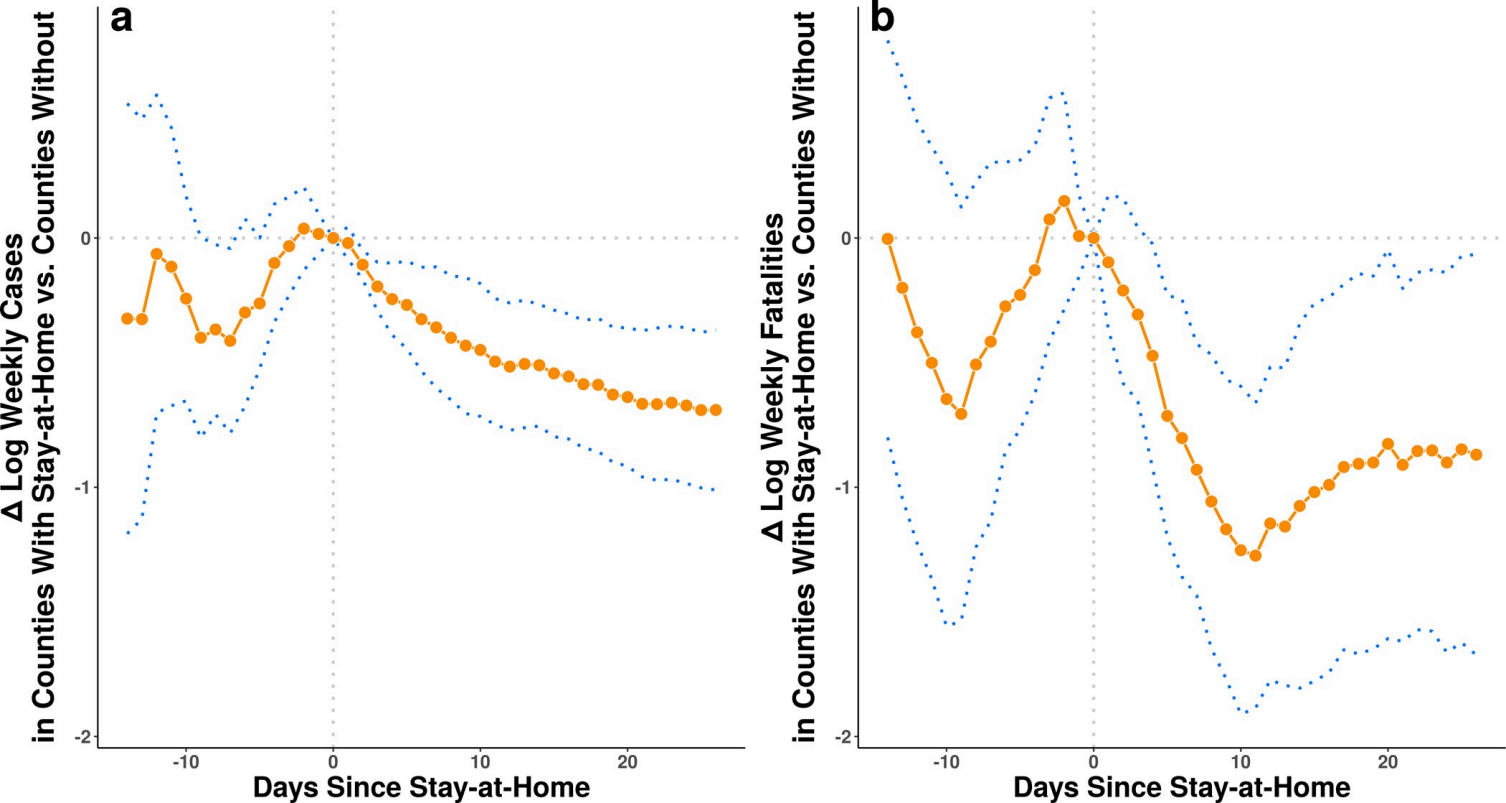
Estimated association between stay-at-home orders and one week change in log weekly confirmed COVID-19 cases (a) and COVID-19 fatalities (b), by the number of full days since the orders were issued. Estimates are derived from fixed-effects regression models that control for county-group level and date fixed effects and for correlated observations with cluster-robust standard errors at the county-group level (see Methods). Blue dotted lines represent 95% confidence intervals.

Fig 4 shows estimated coefficients from the difference-in-differences model for change in case (panel a) and fatality (panel b) growth on each day before and after the day a stay-at-home order goes into effect. The prior day estimates serve as a placebo test to see if counties that issue orders have differential case growth relative to counties that do not issue an order in the days prior to the order.

Unlike the raw data shown in Tables 2 and 3, the estimates here are adjusted for unobserved factors that could influence the course of the disease that vary over time and between counties. Notice that the point estimates in Fig 4 before the order goes into effect exhibit variability with confidence intervals that span zero. This suggests that county-group-level differences in case and fatality growth are not strongly related to the timing of stay-at-home orders, helping to rule out the possibility that the later associations we see are driven by reverse-causality or differential trends.

It is important to remember that one unit of a logarithm is an order of magnitude, and as such these results suggest a large association between stay-at-home orders and alterations in cases and fatalities. What were the policy consequences of these interventions? We use the model estimates from Table 3 to estimate cases and deaths for each county group in an alternative world where no orders were issued. Our model estimates how much case growth may have declined in counties that issued orders (see Methods). To estimate cases in a counterfactual setting without orders, we assume that growth in the absence of a stay-at-home order would have followed the trajectory estimated for no-stay-at-home counties by the model. That is, imagine a simple model that estimated case growth was 10% lower in counties with orders. To estimate counterfactual cases, we would take the observed case counts in each county on the day of the order and apply a growth rate 10% lower than observed to estimate what cases would have actually looked like. Our actual model is more complicated than this, estimating different order effects for different county groups, but this represents the basic idea.

Using the coefficient estimates in Table 3, we calculate the expected value of cases for counties that did issue orders where the indicator variable set to zero (no order) rather than one. This is the model-based estimate of the case progression absent an order. We use the upper and lower bounds of the coefficient confidence intervals to calculate upper and lower bounds on the expected counterfactual case counts. These results suggest that stay-at-home orders reduced confirmed cases nationwide by 390,000 (170,000 to 680,000) and fatalities by 41,000 (27,000 to 59,000) within the first three weeks. These estimates suggest reductions in cases of 25% and in fatalities of 35%, respectively. As with all counterfactual projections, our estimates here rely on the estimating assumptions built into our empirical analysis and should be interpreted accordingly.

## Discussion

The results here provide additional evidence that stay-at-home orders in the United States may be effective in limiting the spread of COVID-19 and indicate that physical distancing measures may work to “flatten the curve.” The results also suggest that stay-at-home orders are associated with a dramatic reduction in the number of cases and fatalities that result from the disease.

With that said, we note certain limitations in our analysis. Stay-at-home policies are ultimately assigned endogenously so, as with any observational study, we cannot say for certain that the associations we have measured are the result of a causal effect. Our tests of reverse causality suggest that stay-at-home orders influence case growth and not the other way around, but there is no way around the fact that these are observational data from which causal estimates are notoriously difficult to obtain.

Our dependent variables—cases and fatalities—are based on incomplete data. It is well-known that rates of testing in the United States were extremely low in the early part of the pandemic [2, 25], so measures of cumulative cases and fatalities over time probably increased faster than the disease itself due to the addition of previously undetected infections. And many cases and fatalities may have gone undetected entirely.

Our independent variable, stay-at-home order status, measures a policy intervention that was often implemented simultaneously or within days of several other local interventions, such as bans on mass gatherings and closures of schools, non-essential businesses, and/or public areas. Given the uncertainty about how many days infected individuals are contagious both before and after the onset of symptoms, efforts to generate a sharp estimate of the effects of policies that were implemented within days of each other are difficult. Our analysis suggests these other local interventions might also have an effect on cases and fatalities, as we observe reductions in cases and fatalities just a few days subsequent to stay-at-home orders. In addition, we see significant reductions in the growth rate of cases and fatalities within days of the order. This is in spite of the fact that case identification during the early part of our observations was based on tests that often took a week to be resolved.[2, 24]

With our current empirical approach we cannot perfectly separate the effects of other local interventions from that of stay-at-home orders. This means that our estimates should properly be interpreted as the effect of stay-at-home orders bundled with effects of correlated local interventions. Our controls are the set of counties that did not issue mandatory orders, but in those localities residents may have voluntarily changed their social behaviors. As such, our estimates are the difference between the effects of an average bundle of changes in counties that issued orders relative to an average bundle of changes in counties that did not. Because stay-at-home orders—and other policy interventions—were not assigned in a randomized factorial design and were instead largely used as a bundled intervention, it may be ultimately impossible to isolate empirically the individual effects of each specific intervention on cases and fatalities.

One final limitation is that we assume the effects of stay-at-home orders between localities are independent, but it is likely that significant spillovers exist. Consider the effect of the epidemic in New York City on neighboring counties in New Jersey, Connecticut, and as far away as Rhode Island. Or the effect of Mardi Gras in Louisiana or Spring Break in Florida on a variety of locales throughout the United States. This suggests significant spillover of infections. To the extent stay-at-home orders likewise spill over to other localities—as seems reasonable to assume—we have likely *underestimated* the effect of stay-at-home orders because control counties are partially treated by connections to counties with orders.

It is important to note that COVID-19 cases and fatalities continue to be elevated in many areas in the United States and the pandemic is ongoing at time of publication. Our data are limited to the period under study here. There is much still to be done, and we are hopeful that the work here will help our fellow scientists, policymakers, and the public-at-large to plan for the next steps in managing this disease.

## Methods

### Data

The time and date of county-level “stay-at-home” or “shelter-in-place” orders for each state and locality were aggregated and reported on a web page maintained by the *New York Times* starting on March 24, 2020 [26]. As new orders went into effect, this page was updated. We checked it once daily to update the data through May 7, 2020. In some cases a statewide order was reported with reference to earlier city-level or county-level orders in the state without specifying where they occurred. In those cases, we searched local news outlets to find references to official city and county orders in the state that preceded the statewide order. For each county in each state we recorded the earliest time and date that a city, county, or statewide order came into effect.

County-level data on cumulative COVID-19 confirmed cases and fatalities were also aggregated daily by the *New York Times* [27]. We discarded all observations where cases were not assigned to a specific county (these account for 0.8% of total cases). We retained observations where cumulative cases declined from one day to the next due to official revisions to the counts (0.8% of cases).

Availability of tests for COVID-19 in the United States has not been uniform over the date range of the study [25]. To mitigate the effect of changes in rates of testing on our measure of both confirmed cases and fatalities, we also collected data on and control for the number of tests administered each day. This information is not currently available for each county, but it is available for each state by date from the COVID Tracking Project [28]. When we aggregated observations into county groups we measured the weighted mean of these state-level tests, where the weights are the number of counties from a county group in each state.

### Estimation

In our data, we observe cases and fatalities *y* for each county, indexed by *k* and date, indexed by *t*. We would like to compare counties with orders to those without, so we aggregate cases and fatalities into county groups *c*. Each county group includes all counties that implement stay-at-home policies on the same calendar date. This includes 22 groups of counties that each chose to issue a stay-at-home order on a unique date and one group that never issued a stay at home order. Since both cases and fatalities grow exponentially in our data, we measure the rate of change as the difference in log weekly counts and we add 1 to ensure the log is defined:

$$\Delta y_{ct}=ln(\sum_{k\in c,t\in \{-6,0\}}\;y_{ct}+1)-\;ln(\sum_{k\in c,t\in \{-13,-7\}}\;y_{ct}+1).$$ (1)

Because the population is essentially fixed in each county during this short time period, our measure of the dependent variable is equivalent to changes per capita.

For each of the 22 county groups where an order was enacted (*x*_*c*_ = *1*), we include in the data observations from two periods, one on the date of the stay-at-home order (*p*_*ct*_ = *0*) and one on a date *d* days in the future (*p*_*ct+d*_ *= 1*). For comparison, we also include two observations from the group that had no order (*x*_*c*_ = *0*) on the same dates *t* and *t* + *d*. We consider different values of *d* as the timespan of infection is not known. This yields a total of 88 observations for each scalar value *d* that we consider.

We model the effect of stay-at-home orders with a two-way fixed-effects weighted least squares regression model: [29]

$$\Delta y_{ct}=\alpha_{c}+\tau_{t}+\beta_{1}x_{c}+\beta_{2}p_{ct}+\beta_{3}x_{ct}p_{ct}+u_{ct}.$$ (2)

A strength of this model is that fixed effects *α*_*c*_ control for all time-invariant features of each county group that might drive rates of change in cases and fatalities [30]. For example, each county has its own age profile, socioeconomic status, local health care system, base rate of population health, and date on which a first case of COVID-19 was observed. Additionally, time fixed effects *τ*_*t*_ control for factors that vary over time [30]. For example, case rates could be affected by changes in the availability of testing nationally, in social behaviors influenced by daily events reported in the media, and national-level policies that vary from one day to the next.

Including *β*_*1*_*x*_*c*_ allows us to control for the overall difference between counties that ever had an order and those that did not. Similarly, *β*_*2*_*p*_*ct*_ allows us to control for the overall difference between period 1 and period 0. Including the interaction of these two variables *x*_*ct*_*p*_*ct*_ allows us to estimate *β*_*3*_, the difference in the differences between counties that had an order and those that did not. This estimate captures the causal effect of stay-at-home orders on cases and fatalities under one assumption. The assumption is that counties that implement orders on a specific date would have had similar changes in cases as counties that did implement orders if the implementing counties had not issued the order. This is the standard parallel trends assumption of difference-in-difference models.

Finally, we weight each county group observation by the number of counties and we cluster standard errors *u*_ct_ at the county group level. County groups with more counties have more weight than those with fewer counties. This adjusts the estimated standard errors for unobservable factors correlated within county groups and allows interpretation of the marginal effects at the county level.

We can examine the temporal dynamics of stay-at-home orders by repeating the regression model for different values of *d* days between the date of the order and the later post-treatment period. We can also let *d* be negative to see if there are systematic differences in cases and fatalities between counties with and without orders prior to the date orders go into effect. This allows us to test whether differences in cases and fatalities might cause changes in the date an order is enacted, rather than the other way around. Due to data availability constraints, the full range of possible days that we can model is *d* ϵ {−14, 26}. Estimates for these models for both cases and fatalities are shown in Fig 4.

We also use the regression results to estimate a counterfactual number of cases and fatalities possibly prevented by stay-at-home orders. Since *β*_*3*_ is the estimated difference in change in log weekly counts, the counterfactual difference in unlogged counts is simply [*exp*(*β*_*3*_)−*1*] times the observed weekly count. We calculate this separately for each county group for week 1 using the model where *d* = *7* and we repeat for week 2 (model *d* = *14*) and week 3 (model *d* = *21*), summing over all 22 county groups and all 3 weeks. We incorporate the confidence intervals surrounding *β*_*3*_ in order to produce upper and lower bound for our counterfactual estimates, respectively.
